# Editorial: Inter-organ crosstalk during exercise in health and disease: Extracellular vesicles as new kids on the block

**DOI:** 10.3389/fphys.2023.1180972

**Published:** 2023-03-14

**Authors:** Kenneth Verboven, Ivan J. Vechetti

**Affiliations:** ^1^ REVAL—Rehabilitation Research Center, Faculty of Rehabilitation Sciences, Hasselt University, Diepenbeek, Belgium; ^2^ BIOMED—Biomedical Research Institute, Faculty of Medicine and Life Sciences, Hasselt University, Diepenbeek, Belgium; ^3^ Department of Nutrition and Health Sciences, College of Education and Human Sciences, University of Nebraska-Lincoln, Lincoln, NE, United States

**Keywords:** extracellular vesicle (EV), exercise, myokines, exosomes, microRNA (microRNA)

Within exercise physiology, the study of factors potentially mediating interorgan crosstalk during and after exercise is a fascinating field of research. As exercise activates a plethora of metabolic pathways in several tissues, organs and systems, examining the underlying biological mechanisms contributing to exercise related metabolic benefits is imperative. Since two decades, the skeletal muscle is known to secrete humoral factors into the circulation in response to exercise, originally described as “myokines” by Pedersen et al. (2003). These myokines are now well known and extensively studied in the field of exercise science (Pedersen and Febbraio, 2012). Interestingly, exercise also triggers other metabolic organs to release similar factors arising from the heart, liver, white and brown adipose tissue, and the nervous system (Chow et al., 2022). These “exerkines” (Safdar et al., 2016) have been recognized to comprise an extensive range of biologically active signalling molecules, including cytokines, lipids, metabolites and (noncoding) nucleic acids, as recently reviewed (Chow et al., 2022).

Extracellular vesicles (EVs) and their role as carrier particles for molecular signals became of specific interest in the exerkine field, as EVs are considered (co-)drivers of exercise-induced interorgan crosstalk (Whitham et al., 2018; Vechetti et al., 2021). Differentiated by both their size and nature of vesicular biogenesis, EVs can be primarily classified as exosomes, microvesicles and apoptotic bodies although some overlap does exist between these classifications. EVs may enclose plenty of material, including lipids, proteins and nucleic acids (Théry et al., 2018). Indeed, pioneering EV-related exercise studies have shown an increase in the circulating number of EVs after a single bout of exercise (Brahmer et al., 2019; Frühbeis et al., 2015; Oliveira et al., 2018; Whitham et al., 2018), with recent *in vivo* research estimating about 5% of circulating, tetraspanin-positive EVs to be muscle-derived (Estrada et al., 2022). However, the frequent lack of rigorous characterization, purification and/or quantification of EVs (which ideally requires a combination of multiple methodologies) makes the understanding of the role of EVs in exercise physiology rather hard (Darragh et al., 2021) and argues for standard approaches and reporting on EV-related exercise science. Nevertheless, many points need to be clarified, but as the interest in EVs research from an exercise and health perspective is still growing, many researchers are currently trying to understand the mechanisms involved in generation, cell-specific release and uptake of EVs in both health and disease. We therefore proposed a Frontiers Research Topic to present some novel research on the role of EVs in interorgan crosstalk during exercise in health and disease, which resulted in the current Research Topic of 3 original research papers and 1 review paper.

The review by Nederveen et al. discusses the current understanding of the effects of exercise on EVs. Based on existing literature, this review supports the ability of skeletal muscle tissue to secrete bioactive EVs, although tracking cell-specific origin of systemic EVs remains to be elucidated, as well as what population of EVs (either microvesicles or exosomes) are transporting these bioactive signalling molecules. Although the field of exercise EVs is still in its infancy, several factors potentially influencing EV dynamics during and following acute/chronic exercise have been summarized by Nederveen et al., including exercise intensity, training and health status, concomitantly taking into account methodological heterogeneity (with respect to sample Research Topic and handling, but also isolation, purification and quantification of EVs) among existing studies.

In relation to exercise intensity, Kobayashi et al. examined changes in circulating number and proteomic profile of high-intensity interval (HIIT) exercise-induced EVs in a small set of young healthy, physically active males. HIIT exercise rapidly augmented circulatory EVs in the post-exercise phase (i.e., 30 and 120 min after HIIT, respectively), originating from skeletal muscle, liver and adipose tissue, a finding which was suggested to be related to processing by their target cells or degradation. Proteomic analyses of these EVs indicated a prompt elevation of proteins implicated in coagulation cascades, acid-base homeostasis and antioxidative pathways.

Vann et al. used myobundles, a three-dimensional tissue-engineered model of human skeletal muscle, to investigate changes in sarcoplasmic and secretory microRNA expression (miRNA sequencing), the latter assessed in culture medium derived EVs, in response to exercise-mimetic contractile activity *in vitro.* Their findings showed a differential microRNA expression profile (*n* = 152 microRNAs) between myobundles and myobundle derived EVs, of which some were differentially responsive to chronic low frequency stimulation (miRNA-543, -487b-3p, and -6511-3p) or intermittent high frequency stimulation (miRNA-6511-3p, and -543) between myobundles and EVs. As such, Vann et al. provide novel microRNA targets to which the effects of exercise (training) could be explored in future (human) research.

As exercise is a potent modifier of skeletal muscle EV secretion and content in both health and disease, Vechetti et al. found circulating EVs showed similar morphology, but lower concentration, in individuals with cerebral palsy (CP) compared to typically developed individuals, both at rest and following acute aerobic exercise. Aside from congruent morphology, the skeletal muscle-specific microRNAs miRNA-486 was upregulated in CP, irrespective of the exercise stimulus. miRNA-486 might be an important regulator of satellite cells, thereby affecting extracellular matrix and sarcomerogenesis related genes (such as *Pax-7*) and thus contributing to the known CP-related skeletal muscle alterations.

By proposing this Research Topic, our initial goal was to attract manuscripts focusing on the role of the EVs and their responsiveness to the physiological and metabolic adaptations during exercise (training). Ultimately, despite the body of work collected, we only managed to scratch the surface of this rapidly evolving and interesting field in exercise science. Future studies will bring us closer to unravelling the function of the exercise induced EVs in health and diseases.

## References

[B1] Brahmer A. Neuberger E. Esch-Heisser L. Haller N. Jorgensen M. M. Baek R. (2019). Platelets, endothelial cells and leukocytes contribute to the exercise-triggered release of extracellular vesicles into the circulation. J. Extracell. Vesicles 8 (1), 1615820. 10.1080/20013078.2019.1615820 31191831 PMC6542154

[B2] Chow L. S. Gerszten R. E. Taylor J. M. Pedersen B. K. van Praag H. Trappe S. (2022). Exerkines in health, resilience and disease. Nat. Rev. Endocrinol. 18 (5), 273–289. 10.1038/s41574-022-00641-2 35304603 PMC9554896

[B3] Darragh I. A. J. O'Driscoll L. Egan B. (2021). Exercise training and circulating small extracellular vesicles: Appraisal of methodological approaches and current knowledge. Front. Physiol. 12, 738333. 10.3389/fphys.2021.738333 34777006 PMC8581208

[B4] Estrada A. L. Valenti Z. J. Hehn G. Amorese A. J. Williams N. S. Balestrieri N. P. (2022). Extracellular vesicle secretion is tissue-dependent *ex vivo* and skeletal muscle myofiber extracellular vesicles reach the circulation *in vivo* . Am. J. Physiol. Cell Physiol. 322 (2), C246–C259. 10.1152/ajpcell.00580.2020 34910603 PMC8816621

[B5] Frühbeis C. Helmig S. Tug S. Simon P. Krämer-Albers E. M. (2015). Physical exercise induces rapid release of small extracellular vesicles into the circulation. J. Extracell. Vesicles 4, 28239. 10.3402/jev.v4.28239 26142461 PMC4491306

[B6] Oliveira G. P. Jr Porto W. F. Palu C. C. Pereira L. M. Petriz B. Almeida J. A. (2018). Effects of acute aerobic exercise on rats serum extracellular vesicles diameter, concentration and small RNAs content. Front. Physiol. 9, 532. 10.3389/fphys.2018.00532 29881354 PMC5976735

[B7] Pedersen B. K. Febbraio M. A. (2012). Muscles, exercise and obesity: Skeletal muscle as a secretory organ. Nat. Rev. Endocrinol. 8 (8), 457–465. 10.1038/nrendo.2012.49 22473333

[B8] Pedersen B. K. Steensberg A. Fischer C. Keller C. Keller P. Plomgaard P. (2003). Searching for the exercise factor: Is IL-6 a candidate? J. Muscle Res. Cell Motil. 24 (2-3), 113–119. 10.1023/a:1026070911202 14609022

[B9] Safdar A. Saleem A. Tarnopolsky M. A. (2016). The potential of endurance exercise-derived exosomes to treat metabolic diseases. Nat. Rev. Endocrinol. 12 (9), 504–517. 10.1038/nrendo.2016.76 27230949

[B10] Théry C. Witwer K. W. Aikawa E. Alcaraz M. J. Anderson J. D. Andriantsitohaina R. (2018). Minimal information for studies of extracellular vesicles 2018 (MISEV2018): A position statement of the international society for extracellular vesicles and update of the MISEV2014 guidelines. J. Extracell. Vesicles 7 (1), 1535750. 10.1080/20013078.2018.1535750 30637094 PMC6322352

[B11] Vechetti I. J. Jr Valentino T. Mobley C. B. McCarthy J. J. (2021). The role of extracellular vesicles in skeletal muscle and systematic adaptation to exercise. J. Physiol. 599 (3), 845–861. 10.1113/JP278929 31944292 PMC7363579

[B12] Whitham M. Parker B. L. Friedrichsen M. Hingst J. R. Hjorth M. Hughes W. E. (2018). Extracellular vesicles provide a means for tissue crosstalk during exercise. Cell Metab. 27 (1), 237–251. 10.1016/j.cmet.2017.12.001 29320704

